# Cancer‐Associated Fibroblast‐Induced Remodeling of Tumor Microenvironment in Recurrent Bladder Cancer

**DOI:** 10.1002/advs.202303230

**Published:** 2023-09-24

**Authors:** Ting Liang, Tao Tao, Kai Wu, Lisha Liu, Wuwu Xu, Dewang Zhou, Hu Fang, Qiuxia Ding, Guixiao Huang, Song Wu

**Affiliations:** ^1^ Institute of Urology The Third Affiliated Hospital of Shenzhen University Shenzhen 518116 China; ^2^ Shenzhen Following Precision Medical Research Institute Luohu Hospital Group Shenzhen 518000 China; ^3^ Department of Urology South China Hospital of Shenzhen University Shenzhen 518000 China

**Keywords:** bladder carcinoma, cancer‐associated fibroblasts, recurrence, single‐cell RNA sequencing, tumor microenvironment

## Abstract

Bladder carcinoma (BC) recurrence is a major clinical challenge, and targeting the tumor microenvironment (TME) is a promising therapy. However, the relationship between individual TME components, particularly cancer‐associated fibroblasts (CAFs), and tumor recurrence is unclear. Here, TME heterogeneity in primary and recurrent BC is investigated using single‐cell RNA sequence profiling of 62 460 cells. Two cancer stem cell (CSC) subtypes are identified in recurrent BC. An inflammatory CAF subtype, ICAM1+ iCAFs, specifically associated with BC recurrence is also identified. iCAFs are found to secrete FGF2, which acts on the CD44 receptor of rCSC‐M, thereby maintaining tumor stemness and epithelial‐mesenchymal transition. Additionally, THBS1+ monocytes, a group of myeloid‐derived suppressor cells (MDSCs), are enriched in recurrent BC and interacted with CAFs. ICAM1+ iCAFs are found to secrete CCL2, which binds to CCR2 in MDSCs. Moreover, elevated *STAT3*, *NFKB2*, *VEGFA*, and *CTGF* levels in iCAFs reshape the TME in recurrent tumors. CCL2 inhibition in an in situ BC mouse model suppressed tumor growth, decreased MDSCs and Tregs, and fostered tumor immune suppression. The study results highlight the role of iCAFs in TME cell–cell crosstalk during recurrent BC. The identification of pivotal signaling factors driving BC relapse is promising for the development of novel therapies.

## Graphical abstract

Here, it shows that a predicted communication network exists within the tumor microenvironment (TME) of recurrent bladder carcinoma (BC), with a central role played by the cancer‐associated fibroblasts (CAFs) subtype. The CAFs subpopulation actively interacts with other constituents of the TME, including cancer stem cells (CSCs) and myeloid‐derived suppressor cells (MDSCs), thereby shaping immunosuppressive microenvironment that contributes to BC recurrence.

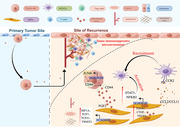

## 1 Introduction

Bladder carcinoma (BC), the most common malignant tumor of the urinary system, is the ninth most common type of cancer worldwide.^[^ ^1^ ^]^ Muscle‐invasive bladder carcinoma (MIBC) accounts for ≈ 25% of all BC cases and is associated with a poor prognosis, significantly affecting patients' quality of life. Non‐muscle‐invasive bladder carcinoma (NMIBC) comprises the remaining 75% of cases and is characterized by a high recurrence rate, ranging from 50% to 70%. Treatment of NMIBC typically involves multiple surgical resections and regular cystoscopic examination, which result in high treatment costs.^[^ ^2^, ^3^ ^]^ However, conventional treatments have limited efficacy due to the high heterogeneity of bladder tumors, resulting in unsatisfactory clinical outcomes. Additionally, there is a close relationship between bladder cancer malignancy, recurrence potential, chemotherapy tolerance, and the tumor microenvironment (TME).^[^ ^4^, ^5^, ^6^ ^]^ Thus, understanding the characteristics of the different components of the TME is crucial for unraveling the mechanisms of BC recurrence and developing novel therapeutic strategies.

A tumor is not merely a simple accumulation of cancer cells; instead, it comprises various cell types that interact intricately with one another to form the TME. The identification of therapeutic targets in cancer biology has been greatly facilitated by the exploration of the TME and the remodeling of the cellular regulatory network.^[^ ^7^ ^]^ However, extensive reprogramming and significant transcriptional heterogeneity of the TME present challenges in the study of different cell subtypes using conventional RNA sequencing techniques, which can only provide information on average RNA expression levels at the whole‐cell level and lack the ability to accurately capture heterogeneity within the sample.^[^ ^8^ ^]^ In 2009, the advent of single‐cell RNA sequencing (scRNA‐seq) technology revolutionized this field. It allows us not only to investigate the characteristics of cancer cells at the single‐cell level, but also to comprehensively depict the functional phenotypes of other cellular components within the TME, including immune cells, fibroblasts, and other stromal cells.^[^ ^9^, ^10^ ^]^ Zhang et al. comprehensively investigated the functional status, composition, dynamics, interaction, and heterogeneity of tumor‐infiltrating T cells and myeloid cells across multiple cancer types using single‐cell transcriptomics, establishing a foundational understanding of the TME heterogeneity.^[^ ^11^, ^12^ ^]^ A recent study employed scRNA‐seq to identify differences in the immune ecosystem between early recurrence and de novo recurrence of hepatocellular carcinoma, providing insight into the heterogeneity of immune‐evasion mechanisms between tumor entities.^[^ ^13^ ^]^ Although BC is known to be one of the least immune‐infiltrating cancers, which may account for its poor response to anti‐PD1 treatment,^[^ ^14^ ^]^ exploring the characteristics of other cell subtypes within the TME holds promise for the development of novel treatments for BC.

Recent studies have revealed the crucial role of CAFs in tumorigenesis and their potential as targets for anticancer therapy.^[^ ^15^ ^]^ And CAFs undergo morphological and functional transitions in the TME, promote cancer progression, and confer resistance to multiple therapies. ScRNA‐seq has revealed light on the functional heterogeneity of different CAF subtypes, including their roles in immunosuppression, angiogenesis, and matrix remodeling.^[^ ^16^, ^17^, ^18^, ^19^ ^]^ Specifically, CAFs have been shown to adopt myo‐cancer‐associated fibroblast (mCAF) or inflammatory cancer‐associated fibroblast (iCAF) phenotypes, as determined by the expression of the markers RGS5 and PDGFRA in BC. Notably, iCAFs have been linked to tumor progression and poor prognosis.^[^ ^20^, ^21^ ^]^ Moreover, Huang et al. utilized scRNA‐seq approaches to identify a distinctive subset of immunomodulatory CAFs, referred to as mesothelial cell‐derived antigen‐presenting CAFs, which were found to promote regulatory T cell expansion in pancreatic cancer.^[^ ^19^ ^]^ Similarly, Liu et al. reported a functional CAF subset in BC, known as interferon‐regulated CAF, which enhances tumor stemness and mediates chemotherapy resistance by secreting WNT5A and interacting with tumor cells.^[^ ^22^ ^]^ Targeting CAFs has shown the potential to improve chemotherapy sensitivity and reduce tumor recurrence.^[^ ^6^, ^23^, ^24^ ^]^ Nonetheless, the heterogeneity of CAFs between primary and recurrent BC and the underlying mechanisms of BC recurrence are not fully understood.

In this study, we used scRNA‐seq to identify a specific subset of CAFs that is significantly associated with BC recurrence. Further analyses revealed that this CAF subpopulation actively communicated with other constituents of the TME, including cancer stem cells (CSCs) and myeloid‐derived suppressor cells (MDSCs), thereby shaping an immunosuppressive microenvironment that contributes to BC recurrence. These findings provide a novel perspective on the intricate cellular interactions within the TME mediated by distinct CAF subpopulations and offer potential therapies that disrupt CAF‐mediated TME modulation to enhance patient outcomes.

## 2 Results

### 2.1 Single‐Cell Transcriptomic Landscape of BC Tissues

**Figure 1:**
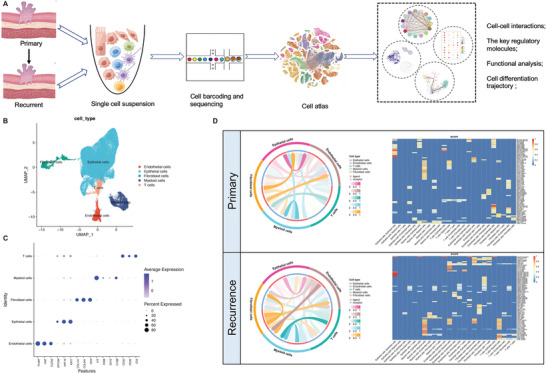
ScRNA‐seq profiling of diverse cell types in primary and recurrent BC. A) Workflow of the specimens collection, processing, and bioinformatic analysis. B) The UMAP plot demonstrates the main cell types in BC. C) Dot plot showing the expression levels of canonical cell markers in each cell type. D) Cellcell communication analysis in primary and recurrent BC by Cellcall.

To systematically survey the TME heterogeneity of primary and recurrent BC, we conducted an in‐depth scRNA‐seq analysis of three primary, four recurrent tumors, and one normal adjacent tissue. Using standard analytical procedures (**Figure** **1**A), we obtained 62460 high‐quality cells from these samples, which were subjected to dimensionality reduction, cell clustering, and identification of five major cell types based on the expression of canonical markers: 51439 epithelial cells, 5070 myeloid cells, 3241 fibroblast cells, 2564 endothelial cells, and 1926 T cells (Figure 1B,C; Figure S1AD and Table S1, Supporting Information). To display distinct subcellular interactions within the TME of primary and recurrent BC, we performed cellcell communication analysis using the CellCall tool (Figure 1D). The results showed a marked increase in intercellular crosstalk events in primary BC compared to recurrent BC. Notably, communication between epithelial and T cells is limited in primary BC, whereas interaction signals between fibroblast and myeloid cells become stronger in recurrent BC, suggesting a potential mechanism underlying immune escape in recurrent tumors.

### 2.2 Heterogeneous CSC Subpopulation Features in Recurrent BC

**Figure 2:**
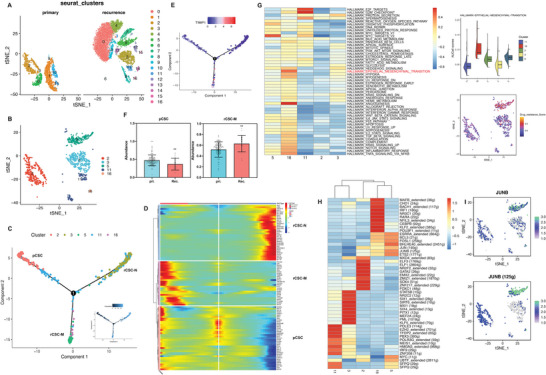
Functional analysis of enriched CSC clusters in primary and recurrent BC. A) The t‐SNE plot shows re‐clustering of epithelial cells in the primary and recurrent BC. B) The t‐SNE plot shows CSC types annotated by cluster numbers. C) Pseudotime analysis of CSC subpopulations inferred by Monocle2. D) Heatmap showing the top100 genes expressed with pseudotime trajectory of three CSC subclusters. E) Trajectory of the marker gene TIMP1 expression. F) The infiltrated abundance of CSC clusters in primary and recurrent BC samples. The results presented are the mean ± SD (n = 159). ^**^, *p*<0.01. G) The pathway enrichment score among different CSC clusters. H) Heatmap showing the area under the curve (AUC) scores of top10 TF motif in CSC clusters by SCENIC. I) tSNE plots of the JUNB expression level (up) and AUC scores (down).

Given that BC is derived from the urinary tract epithelium,^[^ ^25^ ^]^ we characterized the features of the epithelial cells. Compared to normal tissues, epithelial cells in tumor tissues showed abnormal CNV patterns, as was shown by InferCNV analysis (Figure S1E, Supporting Information). In addition, we carried out a Gene Ontology (GO) analysis, which showed that differentially expressed genes (DEGs) in epithelial cells were mainly enriched in calcium binding, cell‐substrate junctions, and focal adhesions between primary and recurrent tumors (Figure S1F, Supporting Information). CSCs are also implicated in cancer recurrence and therapy resistance.^[^ ^26^, ^27^ ^]^ Therefore, we further subdivided the epithelial cells into 17 clusters through dimensionality reduction (**Figure** **2**A), and identified CSC subpopulations in cluster 2 that originated from primary tumor and clusters 3, 5 and 16 originated from recurrent tumor, based on the expression of CSCs markers *CD44*, *ALDH1A1*, and *SOX4* (Figure 2B; Figure S1G, Supporting Information). AddModuleScore analysis revealed cluster 2,3,5 and 16 were stemness (Figure S1H, Supporting Information). Evolutionary trajectory analysis of CSCs from primary to recurrent tumors using Monocle2 revealed three branches, including two types of recurrent‐associated branches: rCSC‐N in cluster 3 and rCSC‐M in clusters 5 and 16, and one primary‐associated branch: pCSC‐N in cluster 2 (Figure 2C,D). Notably, the rCSC‐M subset expressed genes with pseudotime paths similar to those of the pCSC‐N subset, whereas the rCSC‐N subset had a distinct gene expression pattern (Figure 2D). We observed a significant increase in the expression of TIMP1, a hallmark gene involved in metalloproteinase‐mediated extracellular matrix (ECM) remodeling in the rCSC‐M subset,^[^ ^28^ ^]^ as ordered using pseudotime (Figure 2E). Moreover, recurrent BC had higher infiltration of the rCSC‐M subset than primary tumors from the BLCA cohort (Figure 2F). Gene set enrichment analysis revealed remarkable epithelial‐mesenchymal transition (EMT) and drug resistance features in the rCSC‐M subset (clusters 5 and 16) using GSVA and AUCell (Figure 2G). SCENIC analysis also indicated that transcriptional reprogramming occurred in the primary and recurrent CSCs subclusters, including the transcription factors *JUNB* and *BHLHE40*, which are related to drug resistance and the pro‐metastatic phenotype in the TME^[^ ^29^, ^30^ ^]^ with stronger regulatory activity observed in the subclusters of rCSC‐M (Figure 2H,I). These findings indicate a close association between the rCSC‐M subset and BC recurrence.

### 2.3 Specific Functional Phenotype of CAFs in Recurrent BC

**Figure 3:**
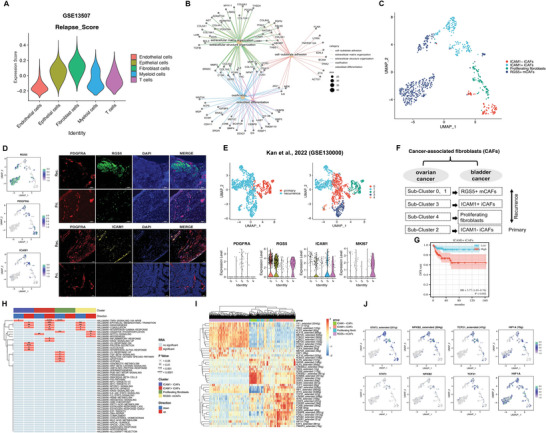
Fibroblast clusters annotation and functional characterization of primary and recurrent BC. A) Identification of relapse‐related signature from published GEO database in diverse cell types. B) Enriched top5 GO_category of DEGs between primary and recurrent BC. C) UMAP plot showing the 4 fibroblast subsets according to different re‐clusters identified. D) UMAP plot and IF confirmed the expression level of the subgroup marker genes. Scale bars, 100 µm. E) UMAP plot showing fibroblasts clusters of published ovarian cancer ScRNA‐seq database (up). Violin plots show the representative markers expression (down). F) Corresponding relationship of different fibroblast subsets among ovarian cancer and bladder cancer. G) KaplanMeier survival curves showing BC patients with low and high infiltration abundance of ICAM+ iCAFs subgroup. H) The pathway enrichment scores by irGSEA per cell between different fibroblast subsets. I) Heatmap of the inferred regulon in each fibroblast subpopulations analyzed by SCENIC. J) UMAP plots showing the expression levels of the representative TF (down) and AUC scores (up).

Based on the gene sets related to relapse in the BC cohort (GSE13507), we focused on fibroblasts (**Figure** **3**A). Subsequent analysis revealed that the DEGs in fibroblasts were predominantly enriched in cell‐substrate adhesion and ECM‐associated pathways (Figure 3B). Re‐clustering of fibroblasts enabled the identification of four distinct subtypes based on markers from previous studies:^[^ ^20^, ^31^ ^]^ ICAM‐iCAFs enriched in primary tumors and ICAM1+ iCAFs and RGS5+ mCAFs enriched in relapsed tumors (Figure 3C; Figure S2AC, Supporting Information). Existing CAF subgroups specific to primary and recurrent tumors were evaluated through immunofluorenscence (IF) staining (Figure 3D). Furthermore, CAF subpopulations with similar characteristics and marker genes were identified in relapsed ovarian cancer (Figure 3E,F).^[^ ^32^ ^]^ Based on the results of the pseudotime trajectory analysis, ICAM1+ iCAFs are likely to be derived from ICAM1‐iCAFs, and ICAM1+ iCAFs demonstrated high expression of genes such as TNFAIP6 and PTGS2 related to the inflammatory response, and AREG, a gene linked to tumor metastasis and drug resistance^[^ ^33^ ^]^ (Figure S2DG, Supporting Information). Functional enrichment analysis revealed that iCAFs were associated with EMT and angiogenesis in both primary and recurrent tumors. However, ICAM1‐ iCAFs were specifically enriched in pathways related to glycolysis and response to interferon‐alpha, whereas ICAM1+ iCAFs showed heightened activity in the inflammatory response, TNFα signaling via the NF‐κB pathway, and response to interferon‐gamma (Figure 3H). Moreover, a higher abundance of ICAM1+ iCAFs was correlated with poor disease‐free survival (DFS) in BC (Figure 3G). Using SCENIC analysis, we identified essential motifs in different fibroblast subgroups, where *NFKB2*, *STAT3*, *TCF7*, *TCF21*, *TWIST2*, and *HIF1A* motifs were highly activated in ICAM1+ iCAFs (Figure 3I,J). Previous studies have shown that *STAT3* and *NFKB2* are drivers of immunosuppressive activation in MDSCs,^[^ ^34^, ^35^ ^]^ whereas *TCF21* and *TWIST2* are associated with EMT.^[^ ^27^, ^36^ ^]^ These findings suggest that the subset of ICAM1+ iCAFs plays a critical role in the recurrence of BC.

### 2.4 Crosstalk between Recurrent BC‐enriched CAFs and CSCs

**Figure 4:**
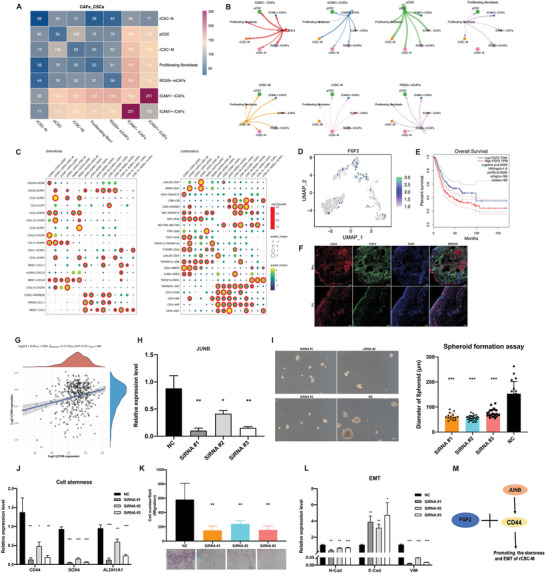
Cellcell communication of fibroblast and CSC subgroups in primary and recurrent BC and functional verification of the potential role of key genes. A) Heatmap of the number of potential ligand‐receptor pairs between fibroblast and CSC subgroups predicted by CellphoneDB. B) Cellcell interactions between each cell subgroup and others. the link size represents the interaction strengthen. C) Bubble plots show ligand‐receptor pairs of chemokines and costimulatory. D) UMAP plots showing the *FGF2* expression level. E) High level of *FGF2* predicted poor prognosis in TCGA BC cohort. F) Detection of CD44 and FGF2 in primary and recurrent tumor tissues by IF staining. Scale bars, 200 µm. G) Correlation between the expression level of *CD44* and *JUNB* in TCGA BC cohort. Coefficient was calculated with spearman correlation analysis, *p* < 0.05. H) qRT‐PCR showing *JUNB* level in UMUC3 cells transduced with siRNA control or siRNA targeting *JUNB*. I) Representative images of colony formation (size) of UMUC3 cells after transduction. Scale bars, 100 µm. Statistical results of the colony formation assay on the right side (n≥15). J) qRT‐PCR showing levels of cell stemness‐related genes after transduction. K) The effect of JUNB on cell migration was examined in UMUC3 cells by transwell filter assay. L) qRT‐PCR showing levels of EMT‐related genes after transduction. The results presented are the mean ± SD (n = 3). ^*^, *p* < 0.05; ^**^, *p*<0.01; ^***^, *p* < 0.001. M) The schematic diagram of regulatory network.

To further elucidate the complex interplay between CAFs and CSCs subpopulations, a comprehensive analysis of cellcell communication was performed, using Cellchat and Cellphonedb. The results demonstrated a strong interaction between specific subpopulations of iCAFs and rCSC‐M and pCSC subsets (**Figure** **4**A,B). Through the visualization of ligand‐receptor pairs involved in cell communication, we determined that ICAM1+ iCAFs recruited rCSC‐M via chemokine signaling pathways (CCL2‐ACKR1 and CXCL12‐CXCR4 axes) and engaged with the ligands CD44 of rCSC‐M via the receptor FGF2 in the costimulatory pathways of recurrent tumors (Figure 4C). Furthermore, we observed a high expression of FGF2 in ICAM1+ CAFs, which was significantly correlated with poor prognosis (Figure 4D,E). IF staining further confirmed the close proximity of FGF2‐positive and CD44‐positive cells as well as the high expression of FGF2 in recurrent tumors (Figure 4F). Previous studies have research has shown that the transcription factor JUNB directly binds to the CD44 promoter sequence and regulates hematopoietic progenitor cell differentiation in vitro.^[^ ^29^ ^]^ Here, *JUNB* motifs were found to be highly activated in the rCSC‐M subset (Figure 2H), and the Spearman correlation of TCGA BLCA cohort indicated a positive correlation between the expression of *JUNB* and *CD44* (Figure 4G). To gain deeper insights into the functions of *JUNB* in BC, we conducted cell functional experiments (Transwell and sphere formation assays) and qRT‐PCR validation of the expression of stemness and EMT‐related genes in JUNB knockdown cell lines using siRNA, revealing that *JUNB* promoted the stemness and migration of tumor cells (Figure 4H,L). Therefore, we hypothesized that JUNB regulates the FGF2‐CD44 signaling pathway and this regulation maintains stemness and promotes EMT of rCSC‐M in recurrent tumors (Figure 4M).

### 2.5 Protumor Phenotypes of Myeloid Cells Subset in Recurrent BC

**Figure 5:**
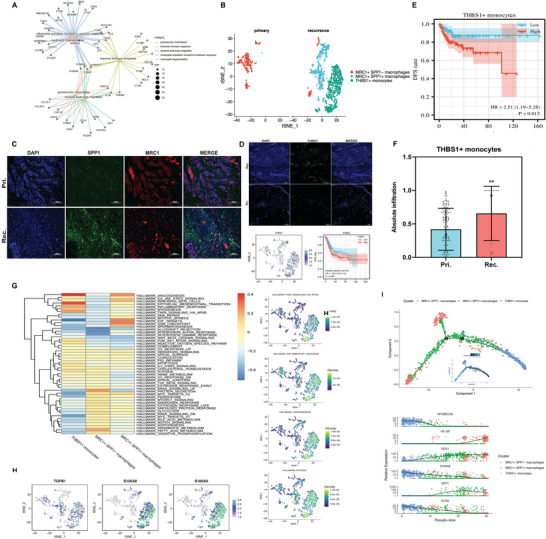
The role of myeloid cells in the immune functions of the recurrent BC. A) Enriched top5 GO_category of DEGs between primary and recurrent BC‐derived myeloid cells. B) tSNE project of myeloid cells, showing the composition of 3 subgroups derived from the primary and recurrent BC. C) IF confirmed the expression level of marker genes SPP1, MRC1. Scale bars, 100 µm. D) IF staining, tSNE plots and KaplanMeier DSS curves for THBS1 level. Scale bars, 100 µm. E) KaplanMeier survival curves showing BC patients with low and high infiltration abundance of THBS1+ monocytes subgroup. F) The infiltrated abundance of THBS1+ monocytes in primary and recurrent BC samples. The results presented are the mean ± SD (n = 153). ^**^, *p*<0.01. G) Differences in pathway activities among different myeloid cells subtypes. H) Feature plots showing the normalized expression of related factors secreted by MDSCs. I) Plot showing pseudotime ordering of different myeloid cells subtypes by Monocle2.

To gain a deeper understanding of the functional roles of CAF subpopulations in the tumor immune microenvironment, we focused on myeloid cells. GO enrichment analysis revealed that the DEGs were mainly associated with granulocyte chemotaxis, humoral immune responses, and myeloid leukocyte migration in primary and recurrent tumors, indicating their potential involvement in regulating immune cell infiltration in the TME (**Figure** **5**A). Subsequently, we further sub‐clustered the myeloid cells and identified three distinct subpopulations based on previous studies:^[^ ^20^, ^31^ ^]^ MRC1+ SPP1‐ macrophages enriched in primary tumors, MRC1+ SPP1+ macrophages, and THBS1+ monocytes enriched in recurrence (Figure 5B; Figure S3A,B, Supporting Information). High expression levels of SPP1 and MRC1 were confirmed by immunofluorescence IF staining in recurrent tumors, whereas THBS1, which has been reported to promote migration of malignant cancers,^[^ ^37^ ^]^ was predominantly expressed in recurrent BC and was found to be a predictor of poor prognosis (Figure 5C,D). We further confirmed the higher infiltration abundance of THBS1+ monocytes in recurrent samples using a BC cohort and their significant association with poor DFS prognosis (Figure 5E,F). In contrast, THBS1+ monocytes showed enrichment of TNFA‐via‐NFκB signaling pathways, inflammatory response, angiogenesis and hypoxia induction when analyzed by GSVA and AUCell (Figure 5G). The myeloid cell subset also expressed high levels of the genes *TGFB1*, *S100A8* and *S100A9*, which have been reported to be favorable for the formation of pre‐metastatic niches mediated by MDSCs (Figure 5H).^[^ ^38^, ^39^ ^]^ The pseudotime trajectory analysis of myeloid cells revealed that the THBS1+ monocytes subset had the lowest pseudotime score, indicating an initial phase, and showed higher expression of *APOBEC3A*, *VCAN*, *FTH1*, and *FCN1* (Figure 5I; Figure S3C,D, Supporting Information). Therefore, THBS1+ monocytes may function as MDSCs that are involved in tumor immune suppression.

### 2.6 Crosstalk between Recurrent BC‐enriched CAFs and Myeloid Subsets

**Figure 6:**
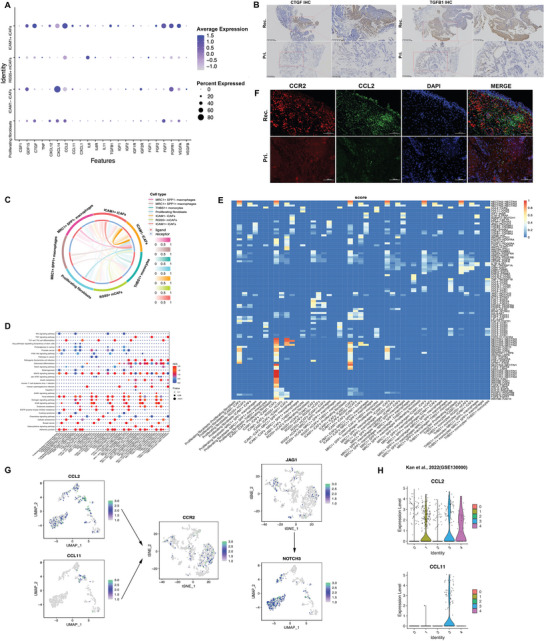
The role of fibroblast clusters in cell communication with myeloid cells subtypes. A) Dot plot shows the expression level of cytokines across fibroblast subtypes. B) IHC staining show expression level of CTGF and TGFB1 in primary and recurrent sample. Scale bars, 400 µm or 200 µm. C) cellcell communication between fibroblast and myeloid cells subtypes by CellCall. D) Bubble plot showing the pathway activity scores of cell interactions. E) Heatmap plot of cellcell communication scores for ligand‐receptor pairs. F) IF staining shows the representative CCL2‐CCR2 pair levels in primary and recurrent sample. Scale bars, 100 µm. G) Feature plots showing the expression levels of representative ligand‐receptor pairs. H) Violin plot indicating the expression of potential marker genes in fibroblasts clusters of published ovarian cancer ScRNA‐seq database.

In previous studies, iCAFs were identified as the primary source of cytokines in BC.^[^ ^20^ ^]^ To investigate the expression levels of various cytokines, we examined ICAM1+ iCAFs in recurrent tumors and found that *CCL2*, *IL‐6*, *TGFβ*, *CTGF*, *FGF2*, *FGF7*, and *VEGFA* were significantly enriched (**Figure** **6**A). Furthermore, immunohistochemistry (IHC) staining revealed that two ECM remodeling‐related genes, CTGF and TGFB1, were markedly upregulated in recurrent BC (Figure 6B). Using NicheNet analysis to identify the top‐ranked ligands that regulate CAFs in THBS1+ monocytes, we discovered that the representative enrichment pathways of the predicted target genes expressed in CAFs were associated with VEGFA‐VEGFR2 signaling and ECM organization (Figure S4, Supporting Information). Notably, the chemokine CCL2 recruits myeloid cells to the tumor stroma and promotes immune evasion.^[^ ^40^, ^41^ ^]^ To further investigate the interaction between CAFs and myeloid cell subpopulations, we performed cellcell communication analysis using CellCall and found that the interaction between ICAM1+ CAFs and THBS1+ monocytes was enriched in the adherens junction and chemokine signaling pathway (Figure 6C,D). As illustrated in a heatmap plot of communication scores for ligand‐receptor pairs, ICAM1+ iCAFs interacted with THBS1+ monocytes through the CCL2/CCL11 receptor and CCR2 ligand, RGS5+ mCAFs interacted with THBS1+ monocytes through the CCL2‐CCR2 axis, and THBS1+ monocytes interacted with RGS5+ mCAFs through the JAG1‐NOTCH3 axis (Figure 6E). JAG1 mediates the Notch signaling pathway to promote the EMT in CSCs and angiogenesis.^[^ ^42^, ^43^ ^]^ And the CAF and myeloid subgroups in recurrent tumors showed remarkably high levels of signaling molecules, namely *CCL2*, *CCL11*, *CCR2*, *JAG1*, and *NOTCH3* (Figure 6G). IF staining also revealed a higher abundance of CCL2‐positive and CCR2‐positive cells in recurrent tumors (Figure 6F). Furthermore, *CCL2* and *CCL11* were highly expressed in the CAF subclusters associated with recurrence in the ovarian cancer single‐cell dataset (Figure 6G).^[^ ^32^ ^]^ Taken together, these findings suggest that the ICAM1+ iCAFs may recruit MDSCs (THBS1+ monocytes) to mediate immune suppression in recurrent cancers via the CCL2‐CCR2 signaling axis.

### 2.7 The Roles of CCL2 Blockade to Tumorigenesis and Immunosuppressive Characteristics in Recurrent BC

**Figure 7:**
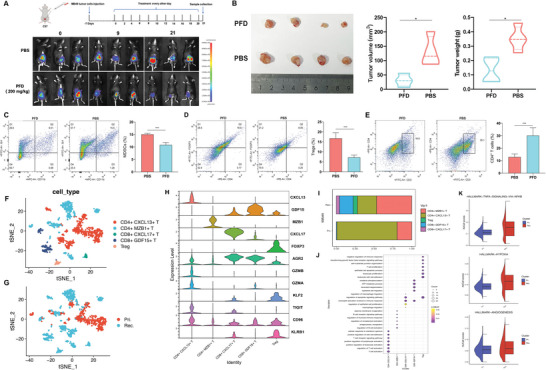
CCL2 contributes to tumorigenesis and immunosuppressive characteristics in recurrent BC. A) In vivo bioluminescence imaging of C57 mice bearing MB49 cells co‐inoculated tumor treated with CCL2 inhibitor (PFD) and control (PBS). B) Representative tumor pictures were presented, and tumor volume and tumor weight at the endpoint were measured (n = 4). ^*^, *p* < 0.05. Flow cytometry assay and quantitative analysis of tumor‐infiltrated MDSCs C) Tregs cells D) and CD4^+^ T cells E) after different treatments. The results presented are the mean ± SD (n = 3). ^***^, *p* < 0.001. Feature plots of T cells, colored by the identified cell subpopulations F) and tumor origin G). H) Violin plots show expression level of marker genes across T cell subtypes. I) Average proportion of T cell subtypes in primary and recurrent BC. J) The potential biological functions of T cell subclusters were evaluated by GO analyses. K) Hallmarker gene sets enrichment analysis of T cells in primary and recurrent tumors.

To validate the carcinogenic effect of CCL2, we employed pirfenidone (PFD), a CCL2 inhibitor, to treat MB49 cell‐C57BL/6 J mouse bladder orthotopic tumor models. Our findings demonstrated that PFD‐treated mice exhibited a reduced tumor burden, as evidenced by lower tumor volume and weight at the endpoint compared to the control group given phosphate‐buffered saline (PBS) (**Figure** **7**A,B). Additionally, flow cytometry analysis showed a decreased abundance of MDSCs and Tregs infiltrating the tumors, whereas CD4+ T cell abundance increased in the treated group compared to the control group (Figure 7C,E). To further investigate the changes in T cells in recurrent tumors, T cells were sub‐clustered and annotated into five subpopulations (Figure 7F,G). The abundance of Tregs was significantly increased in recurrent tumors, whereas the proportion of CD4+ T cells was decreased, consistent with our flow cytometry analysis. Moreover, recurrent tumors showed higher infiltration of CD8+ T cells (Figure 7I). The abundance of CXCL13+ CD4+ T cells, the tumor antigen‐specific T cells to immune‐checkpoint blockade,^[^ ^44^, ^45^ ^]^ was significantly higher in primary tumors, and a subset of CD4+ T cells had high expression levels of several cytotoxic genes, including *GZMB* and *GZMK* (Figure 7H,I). Additionally, *GDF15* is highly expressed in T cells from recurrent tumors, which may be associated with antigen recognition and the inhibition of anti‐tumor immune responses.^[^ ^13^ ^]^ *ARG2*, an immune inhibitory molecule, is highly expressed at high levels in CD8+ T and Treg cells. We also assessed the expression of immune checkpoint receptors in each T cell subpopulation and found that the expression of *CD96* and *KLRB1*(*CD161*) was higher in CD8+ T and Treg cells than in CD4+ T cells (Figure 7I). CD161 blockers have been reported to effectively enhance the T cell‐mediated killing of glioma cells and significantly improve antitumor function.^[^ ^46^ ^]^ GO analyses were performed to investigate the possible biological functions and associated signaling pathways of each T cell subpopulation. The CXCL13+ CD4+ T cell cluster was found to be enriched in the positive regulation of lymphocyte activation and cellular responses to interferon‐gamma. Conversely, the T cell subpopulations in recurrent tumors were associated with negative regulation of the immune response and neutrophil activation involved in the immune response (Figure 7J). The scoring of hallmark gene sets revealed that T cells in recurrent tumors reinforced the activity of pathways related to the pro‐inflammatory response and hypoxia induction within the TME (Figure 7K), further suggesting that recurrent BC exhibited higher immunosuppressive features of the TME.

## 3 Discussion

**Figure 8:**
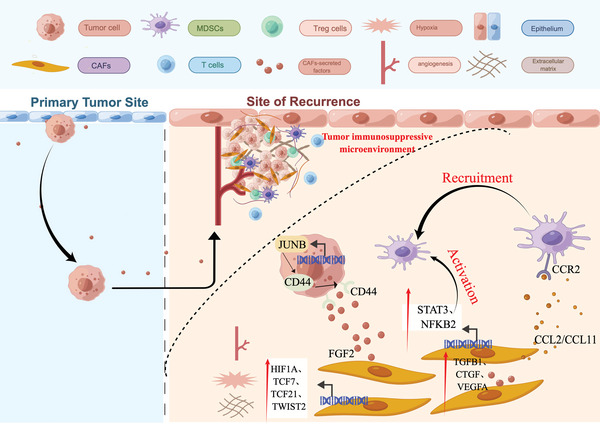
Diagram illustrating the predicted regulatory network of recurrent BC TME centered on iCAF subtype by Figdraw (www.figdraw.com).

The recurrence rate of BC remains a significant obstacle to satisfactory treatment. Despite the remarkable success of immunotherapy, its efficacy is still limited to a fraction of patients, and the underlying mechanisms of non‐response are not yet fully understood. In particular, anti‐immune checkpoint therapies such as PD1/PD‐L1 inhibitors, provide clinical benefits to ≈ 30% of patients with advanced disease.^[^ ^47^, ^48^ ^]^ Thus, identifying novel targets and developing combinatory treatment regimens is imperative to improve treatment outcomes for the majority of patients with recurrent BC. In this study, we performed an integrated analysis of scRNA‐seq data obtained from both primary and recurrent BC samples, which led to the identification of a distinct subpopulation of iCAFs in the TME. Our findings suggest that this particular subset of CAFs is closely associated with CSCs and MDSCs, thereby facilitating remodeling of the TME and ultimately contributing to tumor recurrence (**Figure** **8**). By undertaking critical reprogramming of the TME, as described in our study, we unveiled the underlying mechanism of BC recurrence and provide crucial insights for clinical prevention and treatment.

A strong correlation has been established between CAFs and high rates of tumor recurrence.^[^ ^49^ ^]^ In this study, we conducted a systematic analysis to investigate the heterogeneity and characteristics of CAFs in recurrent tumors. Our results revealed that a subset of CAFs, namely iCAFs, play a crucial role in promoting angiogenesis and EMT, thereby facilitating the generation of new tumors or the migration of primary tumors. Further trajectory analysis suggested that ICAM1+ iCAFs in recurrent tumors might originate from ICAM‐ iCAFs in primary tumors (Figure S2E, Supporting Information). Notably, previous studies have demonstrated the inflammatory phenotype of CAFs in BC, with iCAFs secreting cytokines that promote cancer cell proliferation.^[^ ^20^ ^]^ In our study, we delved deeper into the heterogeneity of iCAFs subsets between primary and recurrent BC based on the expression level of ICAM1, a known prerequisite for immune suppression,^[^ ^50^ ^]^ and found that iCAFs in recurrent tumors exhibited stronger expression of transcription factors and cytokines, including *HIF1A*, *TCF7*, *TCF21*, *TWIST2*, *CTGF*, *TGFB1*, and *VEGFA*, which are responsible for maintaining hypoxia, reshaping the ECM, and enhancing angiogenesis in the TME (Figures 3, 6,6A,8). The results of the present study highlight the significant heterogeneity of the TME across different pathological stages and emphasize the importance of studying the distinct characteristics and functions of different CAF subtypes. As further validation, we utilized the CIBERSORTx algorithm^[^ ^51^ ^]^ to predict the abundance of specific cell types quantified by scRNA‐seq in publicly available datasets (GSE13507). We found that the RGS5+ mCAFs subset was indeed more infiltrated in recurrent tumor patients than in primary tumor patients. However, the ICAM1+ iCAFs subset was less frequently detected in patients with recurrent BC (Figure S2H, Supporting Information), which could be attributed to the limitations of the deconvolution algorithm CIBERSORTx in inferring cell types in bulk samples and the small sample size of patients with recurrent BC (n = 23). Thus, in future studies, researchers should focus on multiple independent BC cohorts with a larger number of patients with recurrence; algorithms such as scAB and Scissor,^[^ ^52^, ^53^ ^]^ can be applied to accurately identify recurrence‐associated subpopulations by integrating single‐cell and bulk RNA‐seq data.

The intricate interplay between tumor cells, stromal cells, and the ECM within the TME is closely associated with tumor immune evasion, metastasis, and recurrence.^[^ ^54^, ^55^ ^]^ In this study, we aimed to investigate the specific ICAM1+ iCAFs subset in recurrent tumors and its signaling communication with CSCs and myeloid cells by constructing a cellcell interaction network through ligand‐receptor binding. Previous studies have reported that calcium‐binding protein transitions mediate communication between CAFs and cancer cells,^[^ ^56^ ^]^ promoting cancer cell survival and migration. Additionally, under the control of the NF‐κB signaling pathway, CD10+ GPR77+ CAFs secrete IL‐6 and IL‐8, thereby maintaining the CSC phenotype through a paracrine mechanism.^[^ ^57^ ^]^ Our study revealed the existence of two CSC subtypes in recurrent bladder tumors, indicating significant heterogeneity among these tumors. This heterogeneity may arise from residual primary tumor cells or the field‐change cancerization effect on the bladder, leading to the generation of multiple new tumors.^[^ ^58^, ^59^, ^60^ ^]^ In a recent study, two distinct types of hepatocellular carcinoma recurrence were distinguished, based on the timing of recurrence (termed true recurrence and de novo cancer), which underscores the heterogeneity of their immune ecosystems.^[^ ^13^ ^]^ In the present study, we found that the ICAM1+ iCAFs subset can secrete FGF2, selectively bind to the receptor CD44 of rCSC‐M, and increase the stemness and migration of recurrent tumor cells. Nevertheless, further validation is essential to achieve a comprehensive understanding of the regulatory mechanisms and signaling pathways involved in the TME. 3D co‐culture models, such as spheroids and organoids, have great potential to offer a superior representation of the biological characteristics of tumors and signaling interactions between cells while maintaining the authenticity of patient tissue.^[^ ^61^, ^62^ ^]^

In this study, a subset of iCAFs that expresses high levels of the chemokine CCL2, known as ICAM1+ iCAFs, was identified and was found to recruit MDSCs (THBS1+ monocytes) to hypoxic tumor areas by binding to the receptor CCR2. Our findings are in line with those of previous studies in which it was demonstrated that the participation of the CCL2‐CCR2 axis in tumor progression is through the activation of different signaling pathways. Specifically, the recruitment of immune‐suppressive cells such as CCR2+ tumor‐associated macrophages (TAMs) or MDSCs to the TME by CCL2 weakens the body's anti‐tumor immune response, creating an immune‐suppressive microenvironment that fosters tumor progression.^[^ ^41^, ^63^, ^64^ ^]^ Therefore, CCL2 may be a promising target for BC treatment. To validate its effectiveness in preventing and treating recurrence, it is necessary to conduct functional testing using in vitro co‐cultured cell lines or mouse models of recurrent tumors. Immunosuppressive characteristics, such as inhibition of T cell expansion and inducement of Treg homing, occurs if MDSCs are stimulated by transcription factors, namely STAT1, STAT3, STAT6, and NF‐κB.^[^ ^34^, ^35^ ^]^ The activation of downstream JAK/STAT3 signaling pathways occurs when CCR2 binds to CCL2.^[^ ^65^, ^66^ ^]^ Our results showed that the ICAM1+ iCAFs subset had a significant enrichment of transcription factors, such as STAT2 and NFKB2, as well as target motif activities (Figure 3I,J). This suggests that this particular subset of iCAFs in recurrent tumors has a dual function of recruiting and activating MDSCs. Furthermore, MDSCs contributed to ECM remodeling and vascular‐related gene expression in a subset of recurrent tumor CAFs, including *ACTA2*, *TMP1*, *MMP2*, and *COL1A2* (Figure S4, Supporting Information). ECM remodeling is a critical process for the formation of fibrous tissue growth zones.^[^ ^67^ ^]^ In addition, the ICAM1+ iCAFs subset exhibits a high level of *CTGF* expression, which plays a critical role in promoting tumor fibrosis and collagen deposition.^[^ ^68^ ^]^ This process leads to the formation of immune‐exclusion physical barriers that prevent the infiltration of chemotherapeutic drugs and T cells. These findings imply that CAFs and MDSCs interact within the TME, creating a supportive microenvironment for tumors in the surrounding tissues, which nourishes the tumors “seeds” for resettlement. In a recent study, fibroblasts and tumor cells were injected into a mouse model lacking immune function using CCL2 to attract MDSCs and TAMs to the tumor site, enabling the tumor to evade immune surveillance.^[^ ^69^ ^]^ Similarly, Su et al. revealed that inhibiting the interaction between FAP+ fibroblasts and SPP1+ macrophages could improve immunotherapy for colorectal cancer.^[^ ^31^ ^]^ Our research has yielded further evidence supporting the potential involvement of the CCL2‐CCR2 axis in BC recurrence and the feasibility of an anti‐tumor strategy targeting intercellular crosstalk.

In the present study, scRNA‐seq analysis was performed in order to investigate primary and recurrent bladder tumors, uncovering the intratumoral heterogeneity within the TME of recurrent BC. By combining the construction of cellcell interaction networks, in vitro cell experiments, mouse tumor models, and data mining of public tumor databases, we unraveled the molecular mechanisms underlying the key CAF subgroups and molecules that contribute to tumor recurrence. Recently, various therapeutic approaches targeting cancer‐associated fibroblasts (CAFs), including the use of specific antibodies and antibody conjugates with photosensitizers, have been documented.^[^ ^70^, ^71^ ^]^ BC exhibits a high affinity for endoscopic therapeutic modalities, and targeting CAFs in situ may yield a more effective outcome in inhibiting tumor recurrence. These findings shed light on the regulatory mechanisms involved in recurrent BC and inform the development of new therapeutic strategies.

## 4 Experimental Section

### Sample Collection and Single‐Cell Suspension Isolation

The bladder samples employed in this study were procured from patients who underwent transurethral resection of bladder tumors at Shenzhen Luohu District People's Hospital, in accordance with the guidelines established by the Ethics Committee Board of the aforementioned hospital (2020‐LHORMYY‐KYLL‐007). Written informed consent was obtained from all participants. After surgery, fresh tissue samples were collected and preserved in the GEXSCOPETM Tissue Preservation Solution (Singleron, Cologne, Germany) for subsequent processing. Tissue samples were initially washed with phosphate‐buffered saline (PBS; Gibco, USA), then cut into small pieces of 1–2 mm, and digested using GEXSCOPETM Tissue Dissociation Solution (Singleron) at 37°C with oscillation for 15 min. Following digestion, the samples were passed through a 40‐µm sterile strainer, and the supernatant was removed by centrifugation at 1000 rpm for 5 min at 4°C. The cell pellet was then resuspended in 1 mL PBS, treated with 2 mL GEXSCOPETM red blood cell lysis buffer (Singleron), and incubated on ice for 10 min to lyse the red blood cells. Lastly, the single‐cell suspension was collected, resuspended in PBS, and subjected to further analysis.

### Single‐Cell RNA Sequencing and Raw Data Preprocessing

In accordance with the Singleron GEXSCOPER protocol, the GEXSCOPER microfluidic chip was utilized to capture single cell suspension, followed by processing with the GEXSCOPER Single‐Cell RNA Library Kit to generate single‐cell gel bead‐in‐emulsions (GEMs) using reverse transcription mix and single‐cell 3′ beads. The GEMs were then subjected to a series of steps, including cDNA fragmentation, adapter ligation, purification, PCR amplification, fragment selection, and quality control. Lastly, the Illumina HiSeq X platform (Illumina, USA) was used for library sequencing with 150‐bp paired‐end reads. Raw gene expression matrix data were generated using the Celescope single‐cell data processing software developed by Singleron (https://github.com/singleron‐RD/CeleScope). Quality control and data filtering were performed using FASTQC (version 0.11.7) and Cutadapt (version 1.17). Next, read alignment to the reference genome, GRCh38, was performed using STAR (version 2.6.1b). FeatureCounts (version 1.6.2) was used to output the gene expression matrix, after which the Seurat R package (version 3.1.1) was used for downstream analysis.

### Clustering and Cell‐Type Annotation

After cell filtering, the count matrix was normalized, and highly variable genes were identified, and the data were scaled using the SCTransform function provided by Seurat (version 3.1.1). To control for potential batch effects, the FindIntegrationAnchors and IntegrateData functions were used to generate a novel matrix with 4000 features. Principal component analysis (PCA) was conducted using the RunPCA function; significant principal components (PCs) were utilized for cell clustering and Uniform Manifold Approximation and Projection (UMAP) or t‐distributed stochastic neighbor embedding (tSNE) were employed for dimensionality reduction. The FindClusters function was used for cell clustering, and the FindAllMarkers function of the Seurat package was used to identify differentially expressed genes (DEGs) between clusters. Parameters for FindAllMarkers included min.pct = 0.25, logfc.threshold = 0.75, and only pos = T. Non‐parametric Wilcoxon rank‐sum test was performed to calculate p‐values, with p‐values < 0.05 after Bonferroni correction considered significant. Established markers were used,^[^ ^22^ ^]^ including epithelial cells (*EPCAM*, *KRT7*, and *KRT13* positive), myeloid cells (*LYZ*, *CD68*, *CD1E*, and *C1QB* positive), fibroblast cells (*DCN*, *COL3A1*, and *COL1A1* positive), T cells (*CD3D*, *CD3E*, and *CD2* positive), and endothelial cells (*PLVAP*, *VWF*, and *CLDN5* positive).

### Functional Enrichment Analysis

The AddModuleScore function of the Seurat package was used to assign gene set scores for each cell type. The FindMarkers function was used to identify DEGs between primary and recurrent tumors. Gene Ontology (GO) analysis was then performed using the ClusterProfiler package (version 4.1.4). To assess differences in pathway activity across each cell type, the Gene Set Variation Analysis (GSVA) package (version 1.30.0) was employed and utilized the LIMMA package (version 3.38.3) to calculate pathway activity differences for each cell type. The R package irGSEA (version 1.22.0) was used to perform gene set enrichment analysis and retrieved the gene sets from the MSigDB package. To comprehensively evaluate the results of the differential analysis and identify significantly enriched gene sets, the robust rank aggregation (RRA) algorithm was applied from the RobustRankAggreg package (version 1.0.4).

### Analysis of Single‐Cell Trajectories

Pseudo‐time analysis was carried out using the R package Monocle2 (version 2.18.0) to align the transcriptomes of cells in differentiation order. The DDRTree approach in Monocle2 was used for dimensionality reduction and cell mapping. To identify crucial genes (with a q value <0.01) that participated in the striking translational relationships among the cell types and clusters, the plot_pseudotime_heatmap function was employed. The Branched Expression Analysis Modeling (BEAM) was used for statistical analysis, and the plot_genes_branched_heatmap function was used to identify genes regulated in a branch‐dependent manner. In addition, the Slingshot function for pseudotime analysis was utilized, a tool developed by Street et.al.^[^ ^72^ ^]^ to infer the lineage differentiation structure and order of the cells.

### Scenic Analysis

To discern dissimilarities among cell clusters determined by transcription factors or their corresponding target genes, the single‐cell regulatory network inference and clustering (SCENIC) package (version 1.1.2.2)^[^ ^73^ ^]^ was utilized on all individual cells. Co‐expression modules were constructed using the motif database (https://resources.aertslab.org/cistarget/) for GRNBoost and RcisTarget to identify regulons. Regulon activity in each cell was scored using the AUCell function.

### CellCell Communication Analysis

To investigate the dynamic interplay between CAFs and myeloid cells, two R packages were leveraged, namely CellCall (v1.16.0)^[^ ^74^ ^]^ and NicheNet (v1.7.0).^[^ ^75^ ^]^ CellCall, a computational tool that integrates ligand‐receptor pairs and transcription factor activity, was used to infer inter‐ and intracellular communication pathways. Additionally, NicheNet was used employed to analyze the regulatory ligand activity of THBS1+ monocytes in the context of CAFs subpopulations and downstream target gene prediction in recurrent tumors. Specifically, the regulatory activity of the ligands was visualized using a Nichenet_output$ligand_activity_target_heatmap. A heatmap representation of the differentially expressed ligands and receptors was generated by calculating the average gene expression within the specified cell types and scaling across the identified subtypes. In addition to the aforementioned tools, CellChat (version 1.1.3)^[^ ^76^ ^]^ and CellPhoneDB2^[^ ^77^ ^]^ a Python‐based computational analysis tools were utilized to perform ligand‐receptor analysis, extract inferred network information, and visualize the results using default parameters.

### Analyses Based on Public Datasets

Raw bulk RNA‐seq data were acquired from the Gene Expression Omnibus (GEO) database (accession number: GSE13507), comprising 165 primary BC samples and 23 recurrent non‐muscle‐invasive tumor samples from 14 patients. Transcriptome data and prognostic information for BC were obtained from The Cancer Genome Atlas (TCGA) using the cBioPortal database (https://www.cbioportal.org/). CIBERSORTx (http://CIBERSORTx.stanford.edu/) was used to determine the relative proportions of different cell types in each sample based on the gene signature of the cell subgroups using the default parameters. Survival analysis was conducted using the survival package (version 3.2‐10), and KaplanMeier survival curves were illustrated using the Survminer package (version 0.4.9), with P‐values obtained using the log‐rank test. Additionally, scRNA‐seq data for epithelial ovarian cancer were obtained from the GEO dataset (GSE130000), which included four primary and two recurrent samples.

### IHC and IF Staining

Tissue sections that had been fixed in formalin and embedded in paraffin were sliced into 5‐µm sections and subjected to deparaffinization and rehydration. For IHC staining, antigen retrieval was performed with 10 mm sodium citrate buffer (pH 6) at 100°C for 10 min, followed by blocking of endogenous peroxidase activity with 0.3% hydrogen peroxide for 10 min prior to diaminobenzidine (DAB) staining. The primary antibody was incubated with the slides overnight at 4°C, and then the secondary antibody conjugated with Horseradish Peroxidase was added for 30 min at room temperature. DAB was used as the chromogen and the slides were counterstained with hematoxylin and imaged. For IF staining, the slides were washed with PBS and blocked with a solution containing 10% PBS and 1% normal goat serum at room temperature for 1 h. The primary antibody was then added to the samples, which were incubated overnight at 4°C. After washing the samples with PBS three times for 10 min each, secondary antibody conjugated with Alexa‐488 or Alexa‐594 was added and incubated at room temperature for 1 h. The stained tissue sections were mounted with DAPI (Servicebio, China; GDP1024) and anti‐fade mounting buffer (Servicebio, China) and imaged using a fluorescence microscope (Nikon Eclipse Ti‐SR, Japan). The antibodies used in this experiment included: anti‐FGF2 (Servicebio, China; GB114762), anti‐CD44 (Servicebio, China; GB112054), anti‐THBS1 (abcam, USA; ab267388), anti‐RGS5 (Proteintech, USA; 11590‐1‐AP), anti‐PDGFRA (abcam, USA; ab203491), anti‐ICAM1 (abcam, USA; ab282575), anti‐TGFB1 (abcam, USA; ab281316), anti‐CTGF (Servicebio, China; GB11078), anti‐SPP1 (Servicebio, China; GB11500), anti‐MRC1 (Servicebio, China; GB113497), anti‐CCL2 (Servicebio, China; GB113497), and anti‐CCR2 (Servicebio, China; GB11326).

### siRNA‐Mediated JUNB Knocking‐Down

To achieve knockdown of JUNB expression, siRNA targeting JUNB was added to serum‐free growth medium and mixed thoroughly with diluted Lipofectamine™ RNAiMAX Transfection Reagent (Invitrogen, Carlsbad, CA), following the manufacturer's instructions. The resulting transfection complex was then added to complete medium containing 10% FBS and antibiotics and mixed thoroughly with the tumor cell lines. The cells were subsequently incubated at 37°C in a CO^2^ incubator for 24–48 h, which effectively reduced target mRNA levels. The efficiency of JUNB knockdown was determined by quantifying target mRNA levels using quantitative real‐time PCR (qPCR) analysis.

### Cellular Function Assays

For the sphere formation assay, the tumor cells were seeded in 6‐well plates that had been pre‐coated with 2% hydroxyethyl methacrylate (P3932 SigmaAldrich) at a density of 20000 cells per well. The cells were then cultured in DMEM‐F12 medium (D6434 SigmaAldrich) supplemented with 1XB27, 0.2 µg mL^−1^ EGF, 0.2 µg mL^−1^ FGF, and 100 mg mL^−1^ penicillinstreptomycin (17 504 044, PHG0311, PHG0026 Fisher Scientific, France) and grown in suspension without adherence. After three passages, images were captured using MoticamX (Motic Europe S.L.U., Barcelona, Spain) and quantified. In addition, a cell migration assay was conducted using cell‐culture inserts with a pore size of 8 µmol L^−1^ that were coated with Matrigel (catalog no. 354 234, BioCoat). 3 × 10^4^ cells were seeded into each insert in 200 µL of serum‐free DMEM, and DMEM supplemented with 20% FBS was added to the lower chamber as a chemoattractant. After 18 h of incubation, the cells were fixed with methanol, stained with 1% crystal violet, and images were then captured and quantified.

### Animal Model Study

All mice used in the study were reared under specific pathogen‐free conditions, and all animal procedures were approved by the Institutional Ethics Committee of Shenzhen Luohu District People's Hospital. Six to eight‐week‐old C57 mice were intravesically injected with 20 µL of PBS containing 1×10^6^ MB49 tumor cells. After one week, the tumor‐bearing mice were randomly allocated into two groups (58 mice/group) and administered PBS or 200 mg kg^−1^ pirfenidone (PFD) via intraperitoneal injection every other day. During the course of the experiment, tumor growth was monitored and recorded using a multimode in vivo imaging system (AniView100, BioLight, Guangzhou, China). After 29 days of inoculation, tumor tissue was collected, measured for volume and weight, and then minced and digested with collagenase IV (1 mg mL^−1^, Sigma) and DNase I (1 mg mL^−1^, Roche) in RPMI1640 (Gibco) at 37°C for 1 h. The resulting cell suspension was filtered through a 70 µm cell strainer and subjected to red blood cell lysis. The cells were subsequently incubated with fluorochrome‐coupled antibodies at 4°C for 20 min, washed twice with cold PBS, and analyzed using a BD Canto II flow cytometer. FlowJo software was used for data analysis. The antibodies used in this experiment were PE/Cy7‐anti‐CD45 (Proteintech, USA; PE‐65087), APC‐anti‐CD11b (Proteintech; APC‐65055), FITC‐anti‐Gr1(Proteintech; FITC‐65140), PE‐anti‐CD4 (Proteintech; PE‐65104), CoraLite Plus 488 Anti‐Foxp3(Proteintech; CL488‐65089), and FITC‐anti‐CD3(Proteintech, USA; FITC‐65060).

### Statistical Analysis

The statistical graphs and corresponding analyses were performed using GraphPad Prism 8 software (version 8.1.2). Data were presented as means ± standard deviation. Statistical significance was determined using an unpaired Student's *t*‐test to assess the differences between groups. Spearman's correlation analysis was used to examine the relationships between the variables. Survival data analysis was conducted using the KaplanMeier method and log‐rank test. The optimal cutoff value was used to define the gene expression levels for survival analyses in this study. All other statistical analyses were performed using R software (version 4.0.3). Statistical significance was defined as *p* < 0.05.

## Author Contributions

T.L., T.T., and S.W. designed and supervised this study. H.F., Q.D., and G.H. collected clinical samples. T.L., K.W., and W.X. analyzed the scRNA‐seq data. L.L. and D.Z. prepared the single‐cell suspensions. T.L., L.L., and D.Z. performed the IHC and IF staining and cell and animal experiments. T.L. and T.T. prepared the figures, wrote the manuscript, discussed, and reviewed the results.

## Conflict of Interest

The authors declare no conflict of interest.

## Supporting information



Supporting Information

## Data Availability

Data supporting the findings of this study are available from the corresponding author upon reasonable request.
