# Supplementary material for: Cancer‐Associated Fibroblast‐Induced Remodeling of Tumor Microenvironment in Recurrent Bladder Cancer
Source: Adv Sci (Weinh). 2023 Sep 24;10(31):2303230. doi: 10.1002/advs.202303230 (PMC10625065; doi:10.1002/advs.202303230)
Supplement: Supplementary file 1 — Supporting Information [file ADVS-10-2303230-s001.pdf]

ADVANCED SCIENCE
Open Access

Supporting Information

for *Adv. Sci.*, DOI 10.1002/advs.202303230

Cancer-Associated Fibroblast-Induced Remodeling of Tumor Microenvironment in Recurrent Bladder Cancer

Ting Liang, Tao Tao, Kai Wu, Lisha Liu, Wuwu Xu, Dewang Zhou, Hu Fang, Qiuxia Ding, Guixiao Huang and Song Wu**

Supporting Information

Cancer-Associated Fibroblast-Induced Remodeling of Tumor Microenvironment in Recurrent Bladder Cancer

Ting Liang[1, 3#], Tao Tao[1, 3#], Kai Wu[1, 3], Lisha Liu[1, 3], Wuwu Xu[1, 3], Dewang Zhou[1, 3], Hu Fang[2], Qiuxia Ding[1, 3], Guixiao Huang[1], and Song Wu[1, 2, 3]*

Table S1. Clinical data of bladder carcinoma and non-malignant tissues

Sample ID	Gender	Age	Grade	Stage	Surgery	Number of cells
Pri. _1	M	58	High	pT2N0M0	TURBT[a)]	5588
Pri. _2	M	28	Low	pTa	TURBT	5606
Pri. _3	F	78	High	pT1	TURBT	8476
Rec._1	M	58	High	pT2N0M0	TURBT	5512
Rec._2	M	59	High	pT2N0M0	TURBT	15088
Rec._3	M	59	High	pT2N0M0	RC[b)]	10782
Rec._4	M	61	High	pT3N0M0	RC	4926
Rec._Nor.	M	59	-	-	RC	8262

[a)]**TURBT:** transurethral resection of bladder tumor; [b)]**RC:** radical cystectomy

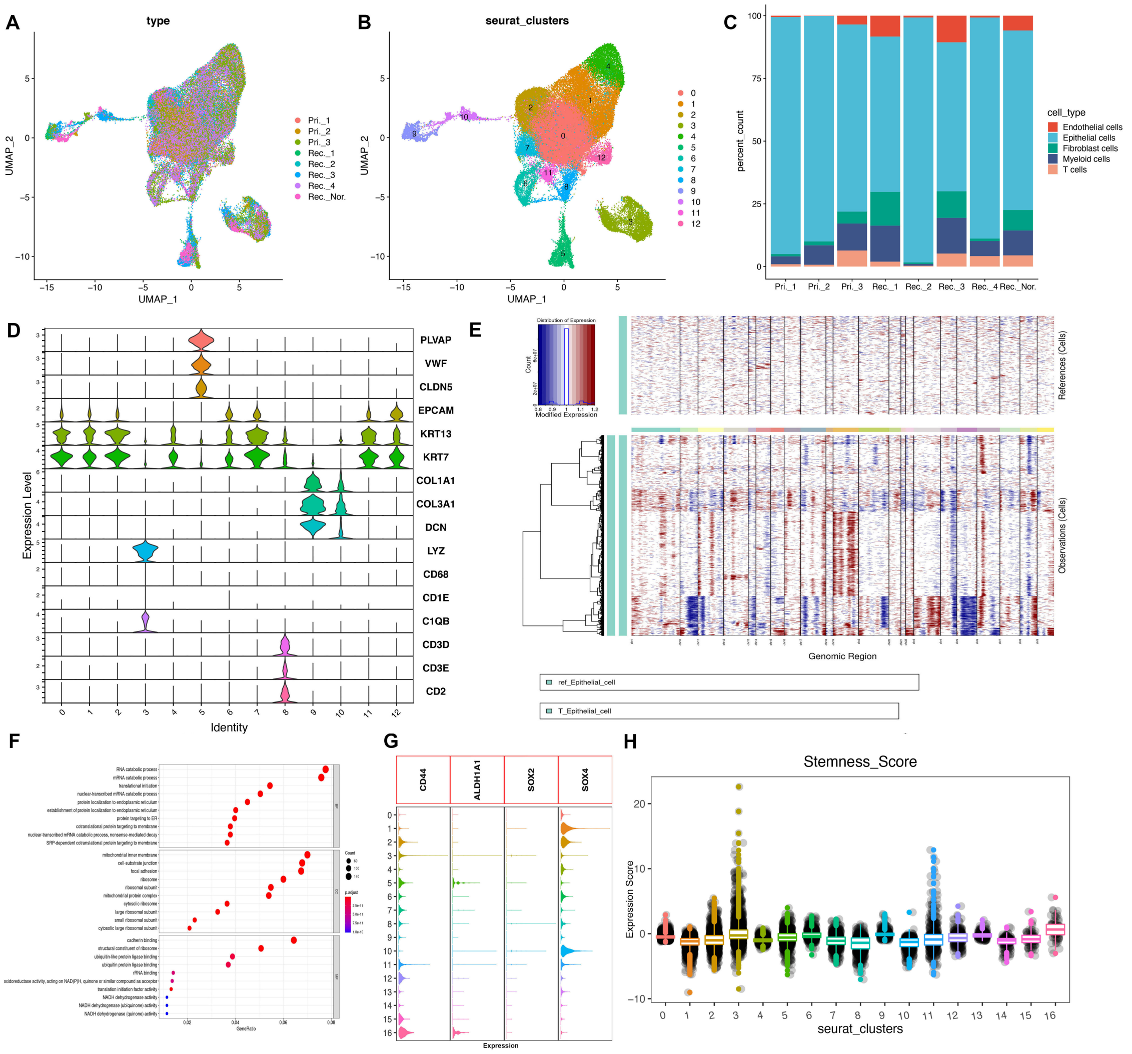

Figure S1. Illustrating the ScRNA-seq profiles of major cell types. A) UMAP plot of single cells profiled here colored by sample origin. B) UMAP plot of single cells profiled here colored by each cluster. C) The fraction of major cell types originated from 3 primary tumors, 4 recurrent tumors and 1 normal adjacent sample. D) Expression of marker genes for each cluster. E) Copy number variations (CNVs) evaluated per cell by InferCNV. Normal-derived epithelial clusters were used as control group. F) Enriched GO functions of DEGs in epithelial cells between primary and recurrent BC. G) Violin plots show expression level of CSC markers. H) The stemness score among different clusters of epithelial cells.

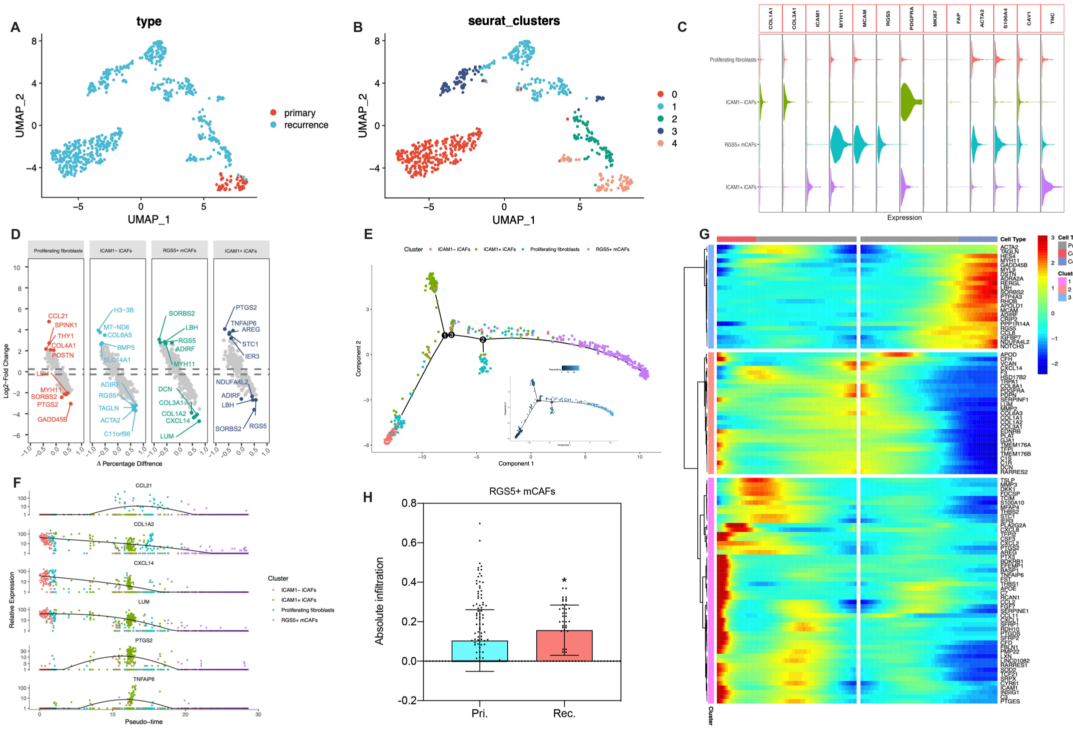

Figure S2. Assessing the functional states of fibroblast cells in primary and recurrent BC. UMAP plot of fibroblast cells, colored by tumor origin (A) and cluster (B). C) Violin plots show expression level of fibroblast subgroups markers. D) Top5 DEGs of different fibroblast subgroups between primary and recurrent BC. E) and G) Plot showing pseudotime ordering of different fibroblast subgroups by Monocle2. F) Trajectory of the representative markers' expression. G) Heatmap showing the top100 genes expressed with pseudotime trajectory of fibroblast subgroups. H) The infiltrated abundance of RGS5+ mCAFs in primary and recurrent BC samples. The results presented are the mean ± SD (n=189). *, P<0.05.

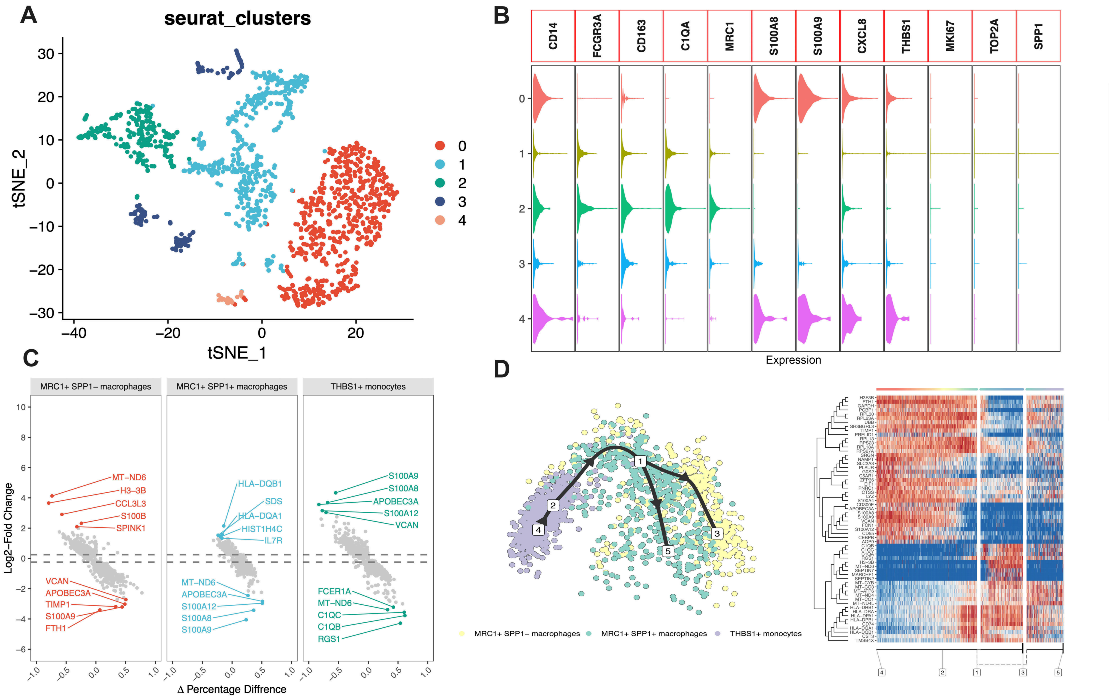

Figure S3. Assessing the functional characteristics of myeloid cells in primary and recurrent BC. A) tSNE plots of myeloid cells, colored by re-clusters. B) Violin plots show expression level of myeloid cells subgroups markers. C) Expression of top5 differential genes in myeloid cells subgroups. D) Plot showing pseudotime ordering of different myeloid cells subtypes by Slingshot, and Trajectory of the top60 markers expression.

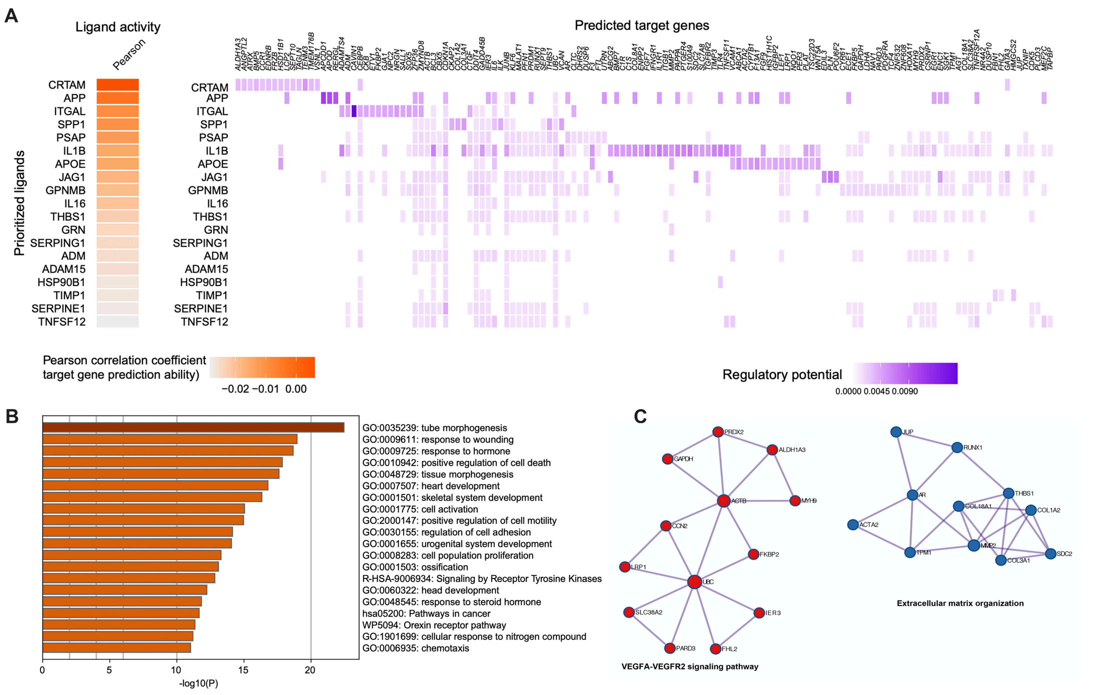

Figure S4. The interaction network between MDSCs and CAFs. A) Top-ranked ligands inferred to regulate CAFs by THBS1+ monocytes and heatmap showing regulatory potential across CAFs according to NicheNet. B) and C) Representative pathways enrichment and Co-expression network of the predicted target genes expressed in CAFs by Metascape.
